# Religious Affiliation Modulates Weekly Cycles of Cropland Burning in Sub-Saharan Africa

**DOI:** 10.1371/journal.pone.0139189

**Published:** 2015-09-29

**Authors:** José M. C. Pereira, Duarte Oom, Paula Pereira, Antónia A. Turkman, K. Feridun Turkman

**Affiliations:** 1 Centro de Estudos Florestais, Instituto Superior de Agronomia, Universidade de Lisboa, Lisbon, Portugal; 2 Escola Superior de Tecnologia de Setúbal, Instituto Politécnico de Setúbal, Setúbal, Portugal; 3 Centro de Estatística e Aplicações, Faculdade de Ciências da Universidade de Lisboa, Lisbon, Portugal; Tulane University School of Public Health, UNITED STATES

## Abstract

Vegetation burning is a common land management practice in Africa, where fire is used for hunting, livestock husbandry, pest control, food gathering, cropland fertilization, and wildfire prevention. Given such strong anthropogenic control of fire, we tested the hypotheses that fire activity displays weekly cycles, and that the week day with the fewest fires depends on regionally predominant religious affiliation. We also analyzed the effect of land use (anthrome) on weekly fire cycle significance. Fire density (fire counts.km^-2^) observed per week day in each region was modeled using a negative binomial regression model, with fire counts as response variable, region area as offset and a structured random effect to account for spatial dependence. Anthrome (settled, cropland, natural, rangeland), religion (Christian, Muslim, mixed) week day, and their 2-way and 3-way interactions were used as independent variables. Models were also built separately for each anthrome, relating regional fire density with week day and religious affiliation. Analysis revealed a significant interaction between religion and week day, i.e. regions with different religious affiliation (Christian, Muslim) display distinct weekly cycles of burning. However, the religion vs. week day interaction only is significant for croplands, i.e. fire activity in African croplands is significantly lower on Sunday in Christian regions and on Friday in Muslim regions. Magnitude of fire activity does not differ significantly among week days in rangelands and in natural areas, where fire use is under less strict control than in croplands. These findings can contribute towards improved specification of ignition patterns in regional/global vegetation fire models, and may lead to more accurate meteorological and chemical weather forecasting.

## Introduction

Vegetation burning displays spatial and temporal patterns at various scales [1,2,3] affected by the seasonal occurrence of dry periods and thunderstorms, and by sporadic heat waves and droughts [4,5]. Land management practices, which often rely on fire, also influence those patterns, timing its use according to crop calendars and ecoclimatic constraints [6]. African vegetation fires are mostly anthropogenic, used for hunting, livestock husbandry, pest control, facilitating food gathering, managing agricultural plot fertility, and for reducing the risk of large, uncontrolled fires [7]. Although the annual and daily dynamics of African biomass burning have been analyzed [8], the occurrence of intermediate scale temporal patterns is undocumented. Biomass burning, a large source of greenhouse gases and aerosols, constitutes a major factor controlling the inter-annual variability of atmospheric composition [9], and has been analysed at time scales ranging from a million years [10], to millennia [11], centuries [12], years [13], and days [8]. Patterns found in these studies were interpreted as a function both of climate dynamics and human activity. Regular cycles in vegetation burning are found at annual scale, mostly in seasonally dry areas, and at the daily scale as a consequence of underlying cycles in meteorological variables and land management practices. Weekly cycles were detected in aerosols and cloud droplet number concentration in several studies, and models confirm that the difference in emissions between week days and weekends generates this cycle. Since there is no natural forcing with a seven-day period, weekly cycles provide evidence of an anthropogenic fingerprint [14]. A weekly cycle of aerosol optical depth (AOD), observed in the United States (U.S.) and in Central Europe, is stronger in urban sites than in rural sites. A reversed AOD weekly cycle occurs in the Middle East and India, with lower AODs on Thursday and Friday, the weekend for Middle Eastern cultures. Therefore, cultural practices and the industrial activity schedule of this region are consistent with the observed AOD weekend effect [15]. Chemical weather forecasting models use meteorological and emissions information as input to simulate transport, chemistry, and deposition processes to predict short-term variability of air quality. Good performance of a chemical weather forecast model requires accurate representation of the temporal distribution of natural and anthropogenic emissions. However, some atmospheric chemistry modelling studies have assumed that, contrary to emissions from industry and transportation, biomass burning does not display weekly cycles [14,16]. Considering that vegetation burning in Africa is mostly anthropogenic and used as a land management tool [4,7,17], we tested the hypotheses that there is a day of the week with lower fire activity, and that this day depends on the regionally predominant religious affiliation: Sunday on predominantly Christian areas, and Friday on predominantly Muslim areas. We also tested the effect of regionally dominant land use on significance of the weekly fire activity minima, which is expected to depend on land management intensity. Our analysis is restricted to the two major religions in sub-Saharan Africa, which account for 62.9% (Christians) and 30.2% (Muslims) of the total population, and are the only ones that prescribe a weekly day of rest [18, 19]. African traditional religions are followed by 3.3% of sub-Saharan Africans, while Hindus and Buddhists jointly represent less than 0.3% of the population, 0.2% have other religious affiliations, including Judaism, and 3.2% are not religiously affiliated [20]. Muslims are concentrated in northern hemisphere sub-Saharan Africa, in a belt extending from the southern part of Sudan, in the east, to Senegal and Sierra Leone in the west, while the southern hemisphere is very predominantly Christian, with the exception of the east coast from northern Mozambique to Somalia. Additional information on the geography of religion in Africa, as classified for the purposes of our analysis, is provided in Materials and Methods.

## Materials and Methods

### Religious affiliation, land use, and fire activity spatial data

**Fig 1:**
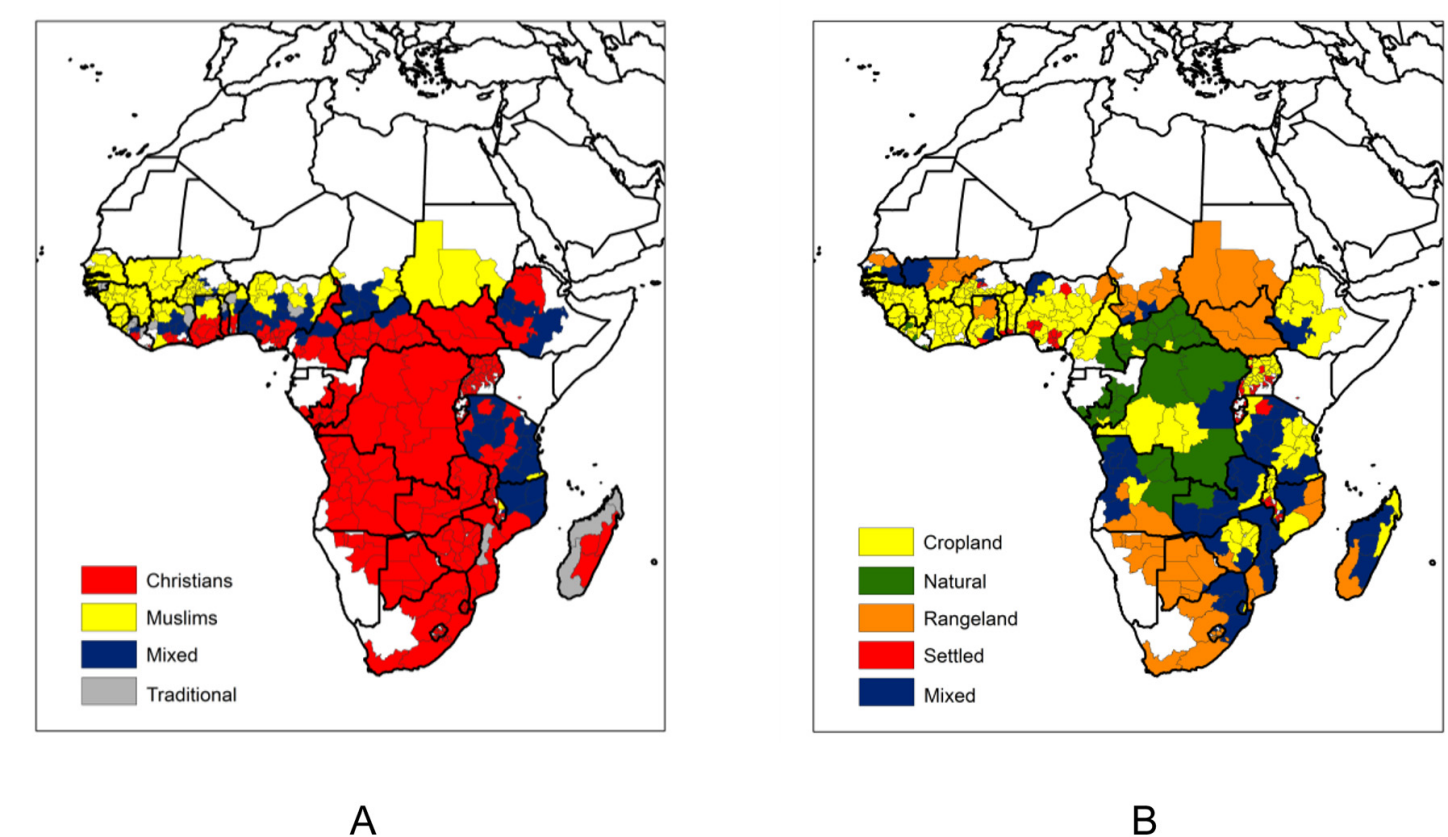
Regional geography of religion and land use in Africa. a) Classification of predominant religious affiliation; b) Classification of predominant anthrome.

The geographical basis for our analysis is the Global Administrative Areas (GADM, version 2, Jan 2012, http://www.gadm.org/) first level divisions for Africa, which contains a total of 554 units and is the spatial support for the religious affiliation data (Fig 1A) of the 2010 World Religion Database [21]. Land use / land cover data (Fig 1B) are from the Anthropogenic Biomes of the World (Anthromes) dataset [22], at 5' (ca. 9 km) spatial resolution and dated from the year 2000. Anthrome data incorporate information on population density, land cover and land use and map global patterns of sustained direct human interaction with ecosystems [23]. Given the strongly anthropogenic character of vegetation burning in Africa they were considered particularly suited for our analysis and preferred over biophysical variables such as vegetation type or land cover. The alternative of using annual land cover data (e.g. the MODIS MCD12Q1 product) would suffer from the interannual classification inconsistencies reported by [24]. The relative stability of spatial patterns depicted by the Anthromes dataset justifies its use to analyze active fire counts from the period 2003–2011.

**Fig 2:**
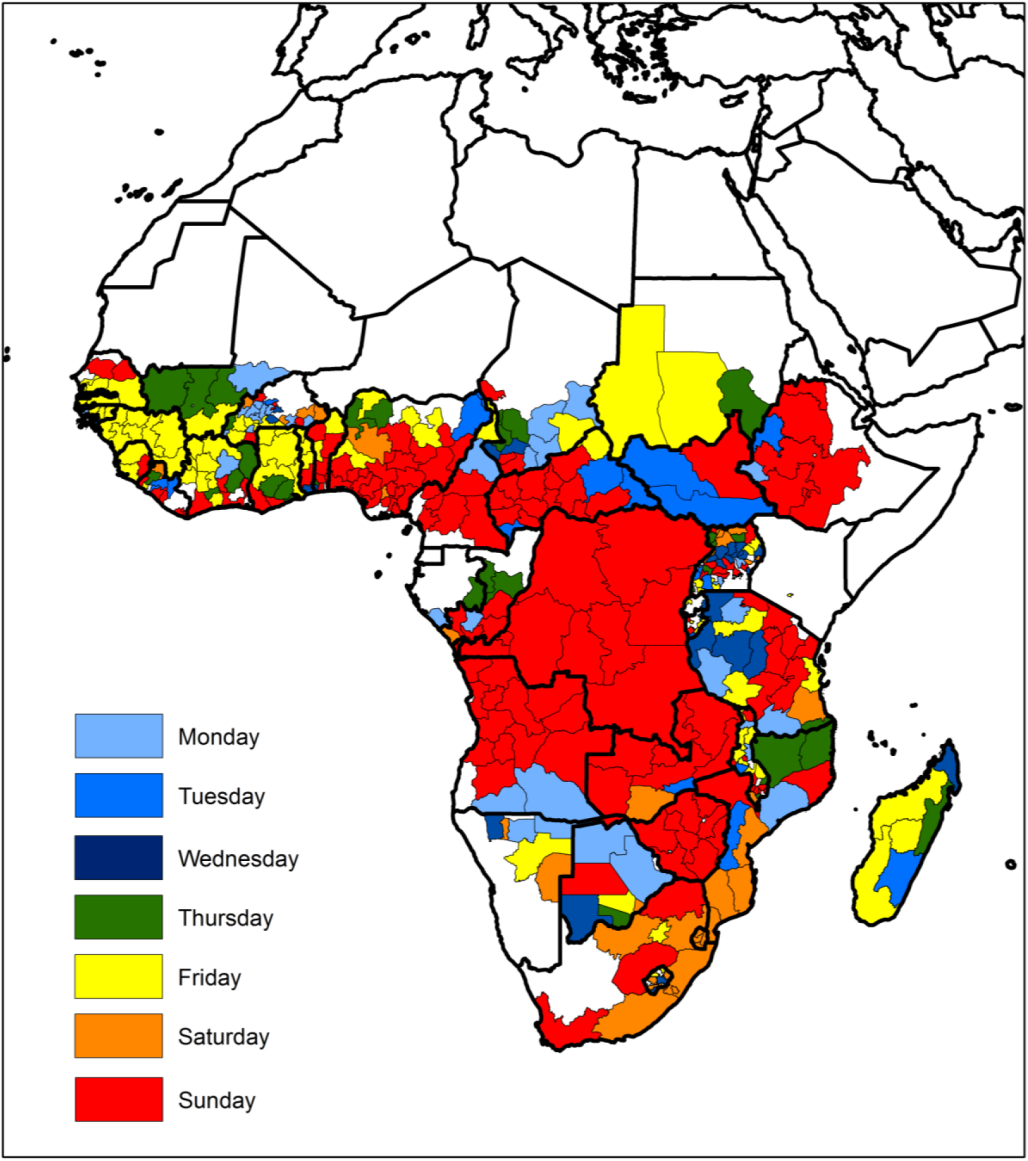
Week day with the fewest fire counts.

**Fig 3:**
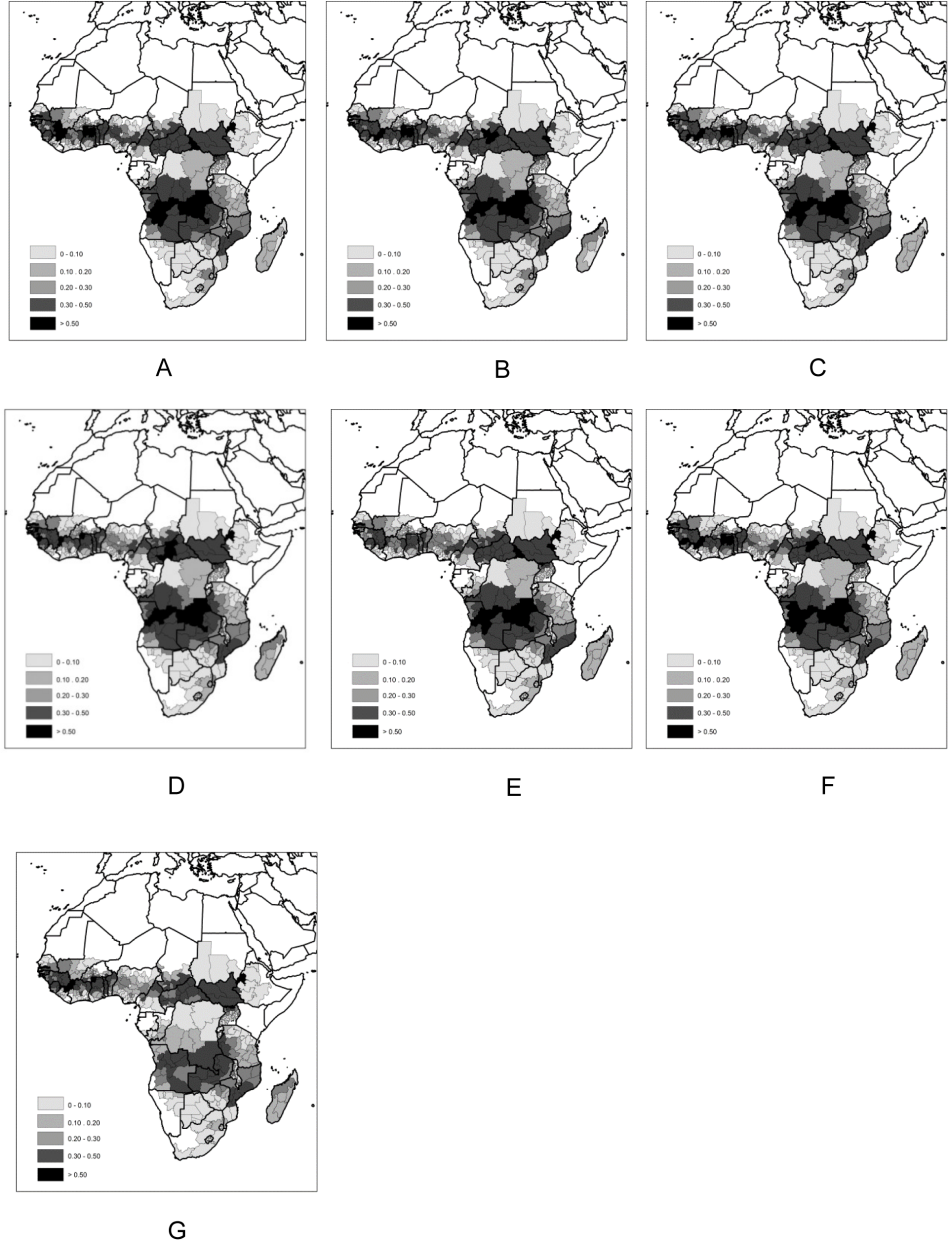
Mean MODIS active fire regional density (fires.km^-2^), 2003–2011. a) Monday; b) Tuesday; c) Wednesday; d) Thursday; e) Friday; f) Saturday; g) Sunday.

Daily fire activity was quantified with data from the NASA MODIS MCD14ML Collection 5 active fire product [25] for the period 2003–2011 (TERRA and AQUA sensors) at 1km spatial resolution, after screening for false alarms and non-vegetation fires according to the procedures detailed in [26]. Fig 2 shows regional fire count densities per week day (Fig 3A–3G) and total density.

### Religious affiliation and land use classification

The screened MODIS active fires dataset contains a total of 22,749,687 observations. One hundred and sixty one GADM regions with less than 0.1 km^-2^ active fire counts accumulated over the nine-year study period were excluded from analysis. An additional nineteen regions where the sum of Christian and Muslim believers falls below 50% of the total population were also removed from analysis, since a weekly day of rest is specific of the Abrahamic monotheisms [18, 19], while in such regions most of the population follows African traditional religions. Two more regions were excluded due to lack of data on religious affiliation, resulting in a total of 372 spatial units analyzed. Wherever Christians (Muslims) represent over 75% of the sum of Christian + Muslim believers, the region was considered Christian (Muslim). When neither religion accounted for at least 75% of the population, the region was labelled mixed Christian-Muslim (Fig 1A). Classification of each region in terms of dominant land use relied on the highest level Anthromes legend [22] with aggregation of the Dense Settlement and Villages classes into a single class (Settled) and the Forest class and Wildland class into a single Natural class. Cropland and Rangeland classes were not altered. The percent area in each region covered by each of the four new classes was calculated and wherever a single class represented at least 50% of the area, the region was labelled as belonging to that class. If no single class exceeded the 50% area threshold, the region was considered as having Mixed land use (Fig 1B). Religious affiliation and Anthrome classification thresholds were selected with the aim of obtaining clear dominance of a class in each region, while ensuring that the respective mixed classes do not cover an unduly large fraction of the study area. Evidently, this was more easily achieved for the three-class religious affiliation variable than for the five class Anthrome variable. Table 1 shows the number of regions by religion and by anthrome, based on these classification rules. Fifty-eight percent of regions (216/372) were classified as Christian and 19% (72/372) as Muslim. Cropland is the anthrome accounting for the highest percentage of regions (164/372, 44%), while the Natural anthrome accounts for the fewest (32/372, 9%).

**Table 1:** Number of regions by dominant religion and dominant anthrome.

	Religion			
Anthrome	Christian	Muslim	Mixed	Total
Cropland	69	47	48	164
Natural	27	0	5	32
Rangeland	30	14	12	56
Settled	48	4	5	57
Mixed	42	7	14	63
Total	216	72	84	372

According to our thresholds for defining predominant religious affiliation, Muslim regions are mostly located in West Africa, in southeastern Chad and in the southern part of Sudan. Most of southern hemisphere Africa is Christian, with the exception of Tanzania and northern Mozambique. Predominantly Christian regions are also found in the southern regions of Ghana, Togo, Benin, Nigeria, and Cameroon, the western part of Central African Republic, South Sudan, and western Ethiopia. Mixed Christian/Muslim regions are located in West Africa, throughout most of Cameroon, in southwestern Chad, the eastern part of Central African Republic and part of Ethiopia, and also in most of Tanzania and northern Mozambique. African traditional religions are dominant in a few regions of West Africa, and in western Madagascar (Fig 1A). Aggregated Anthromes data show that croplands predominate in West Africa, Ethiopia, Uganda, Rwanda, and Burundi, and in parts of the Democratic Republic of Congo, Zimbabwe, and Tanzania. Natural areas are primarily located in central Africa, from the Central African Republic and Cameroon, down to eastern Angola and western Zambia. Rangelands occupy arid areas in the Sahel, and in southern Angola, Namibia, Botswana, and South Africa. Settled regions are concentrated in West Africa, and in the Great Lakes region. Finally, regions of Mixed land use, according to our definition, predominate in southern hemisphere Africa, namely in parts of Angola, Tanzania, Zambia, Mozambique, Madagascar, and South Africa (Fig 1B). Classification of regional fire counts by week day minimum reveals that most of Africa displays a weekly minimum of fire activity on Sunday, especially evident in the southern half of Nigeria, large parts of Cameroon, the Central African Republic, western Ethiopia, the Democratic Republic of Congo, Angola, Zambia, and Zimbabwe. Friday weekly fire minima are primarily found in West Africa, in the southern part of Sudan, and in western Madagascar (Fig 2).

### Statistical modelling

The fire density observed in each region (Fig 3A–3G) was modeled using a negative binomial model [27] with fire counts as response variable, region area as offset and a structured random effect, in the form of a ICAR model [28], to account for spatial dependence. Anthrome, religion and week day, together with their two-way and three-way interactions were used as independent variables. A Bayesian methodology was used to fit these spatial generalized linear model using R-INLA [29]) and the respective package (www.r-inla.org). The best model was chosen using the deviance information criterion (DIC) [30]. For each region *i* = 1, …, 372 characterized by anthrome *A* = 1, …, 5 and religion *R* = 1,2,3, the full model assumes that the total number of fires per year in each week day *W* = 1, …, 7 (*Y*_*iW*_) are conditionally independent observations following the full negative binomial model,

$$P(Y_{iW}=y)=\frac{\Gamma (y+\theta )}{\Gamma (\theta )y!}{\left(\frac{\mu_{iW}}{\mu_{iW}+\theta }\right)}^{y}{\left(\frac{\theta }{\mu_{iW}+\theta }\right)}^{\theta }$$ (Eq 1)

with shape parameter *θ* and mean *μ*_*iW*_ such that:

$$\ln\;\left(\mu_{iW}\right)=\ln\;\left(area_{i}\right)+\alpha_{0}+\alpha_{1A}+\alpha_{2R}+\alpha_{3W}+\alpha_{1RW}+\alpha_{2RA}+\alpha_{3WA}+\alpha_{RWA}+z_{i}$$ (Eq 2)

where *α* is a vector of parameters accounting for the intercept, (*α*_0_) the effect of the three main factors anthrome (*α*_1*A*_), religion (*α*_2*R*_), and week day (*α*_3*W*_), together with the corresponding two-way (*α*_1*RW*_, *α*_2*RA*_, *α*_3*WA*_) and three-way (*α*_*RWA*_) interactions. The area of the region enters in the linear predictor as an offset, ln(area_i_). The term z_i_ is the structured spatial random effect for region *i*, defined by the ICAR model.

To prevent confounding due to association of the anthrome variable both with fire density and religion, the hypothesis of a significant interaction between religion and week day was tested for each of the negative binomial regression models built separately for the different anthromes, taking spatial dependence into account. Again under a Bayesian framework, DIC values of the models were compared with and without the interaction term, and the model with the lowest DIC was selected.

We analyzed specific pairwise contrasts of interest, namely between Sunday and every other week day in Christian regions, and between Friday and every other week day in Muslim regions. The following hypotheses were tested:

$$H_{0}:\mu_{Christian:7}-\mu_{Christian:i}=0,\;\text{for i}=1,2,3,4,5,6;$$

$$H_{0}:\mu_{Muslim:5}-\mu_{Muslim:i}=0,\;\text{for i}=1,2,3,4,6,7;$$

$$H_{0}:\mu_{Christian:7}-\mu_{Muslim:5}=0;$$

$$H_{0}:\mu_{Christian:7}-\mu_{Mixed:7}=0;$$

$$H_{0}:\mu_{Muslim:5}-\mu_{Mixed:5}=0;$$

where 1 = Monday, 2 = Tuesday, 3 = Wednesday, 4 = Thursday, 5 = Friday, 6 = Saturday, and 7 = Sunday. All testing was performed under a Bayesian methodology, by computing, for each of the respective contrasts, the posterior contour probability of zero. The contour probability equals one minus the content of the highest posterior density (HPD) interval just covering zero [31], and is the probability that the posterior density function of the contrast is less than or equal to the posterior density function of the contrast at zero. This probability can be interpreted as a p-value in checking the posterior support of specific values of interest, an approach motivated by well-known analogies between Bayesian and classical inference concepts [32]. To account for multiple comparisons, adjusted values for these probabilities were calculated using the Benjamini-Hochberg method [33]. In addition 95% credible intervals were calculated and displayed.

## Results

**Fig 4:**
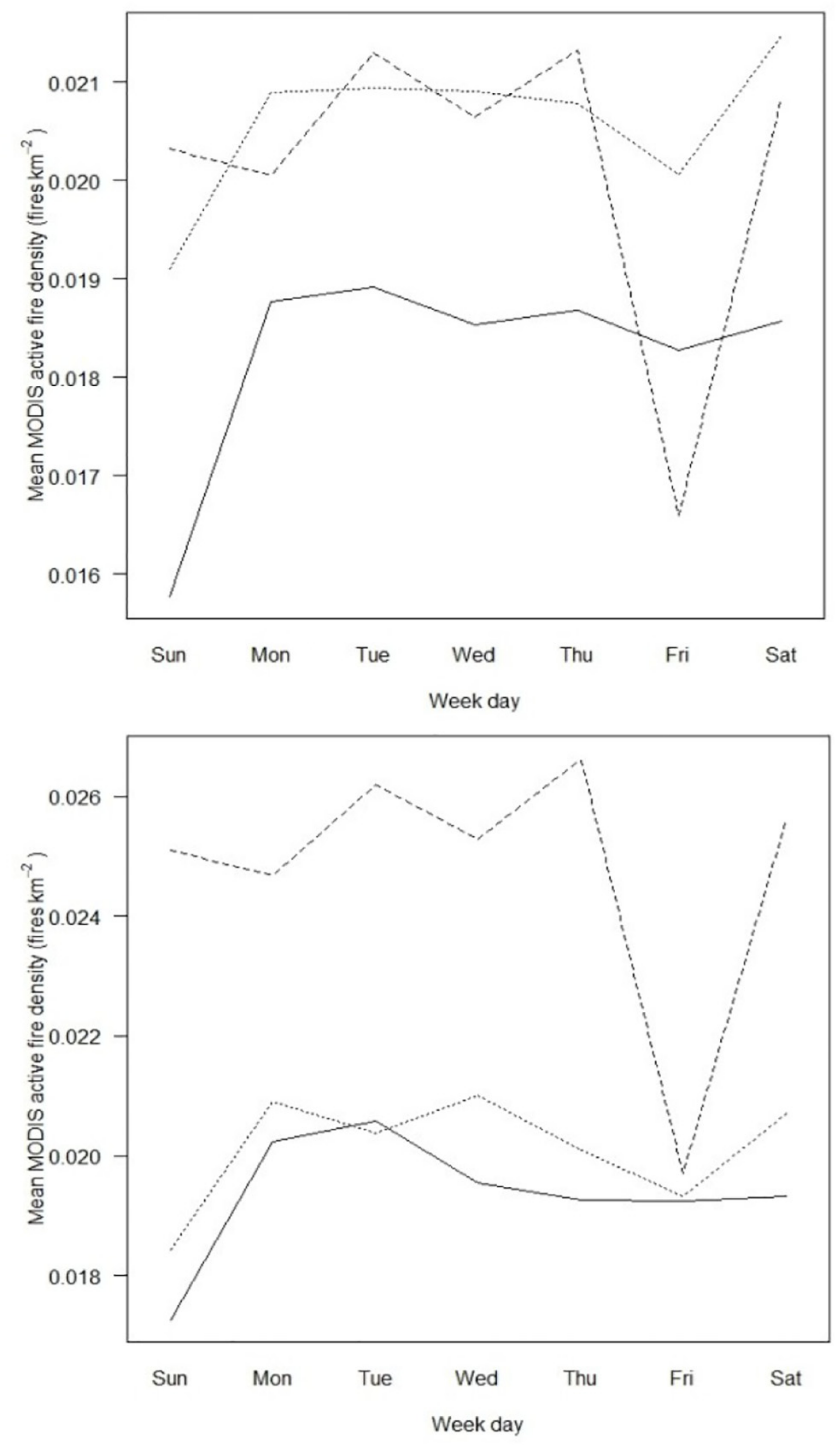
Mean MODIS active fire density (fires.km^-2^) per week day. a) All anthromes; b) Croplands. Solid line: Christian regions; Large dashed line: Muslim regions; Fine dashed line: mixed Christian-Muslim regions.

Statistical models relating regional annual fire counts and regional area with week day of occurrence (nine annual replicates, 2003–2011), religious affiliation, and anthrome, and taking spatial dependence into account, were fit to the data (Table 2). The best model selected using DIC contained the two-way interactions between week day and land use, and between religion and land use, and the spatial random effect in the form of a ICAR model, revealing the existence of a significant interaction between religion and week day, i.e. regions with different religious affiliation (Christian, Muslim, and mixed) display distinct weekly cycles of vegetation burning (Fig 4A). The anthrome variable is strongly associated with religious affiliation and with fire density, acting as a confounding factor in the model. To address this problem, statistical models relating fire density in each region with week day of occurrence, religious affiliation, and the corresponding interaction term were built separately for each anthrome, taking into account spatial dependence. The best model in each anthrome was chosen using DIC. Only for the croplands anthrome (which accounts for 31.2% of the total number of active fires used in the study) did the best model contain the interaction between weekday and religion (Table 3 and Fig 4B).

**Table 2:** Deviance Information Criterion (DIC) scores for all negative binomial models fitted. The best model (lowest DIC, bold) includes all single variables, the two-way interactions between religion and week day, between religion and anthrome, and the spatial term.

MODEL	DIC
R + W + A + R:W + R:A + W:A + R:W:A + ICAR	285 977.17
R + W + A + R:W + R:A + W:A + R:W:A	313 319.00
R + W + A + R:W + R:A + W:A + ICAR	285 936.84
R + W + A + R:W + R:A + W:A	313 252.28
**R + W + A + R:W + R:A + ICAR**	**285 928.25**
R + W + A + R:W + R:A	313 212.31
R + W + A + R:W + W:A + ICAR	285 938.44
R + W + A + R:W + W:A	313 827.29
R + W + A + R:W + ICAR	285 929.88
R + W + A + R:W	313 787.56
R + W + A + ICAR	285 955.56
R + W + A	313 793.07

R–Religion; W–Week day; A–Anthrome; ICAR–Intrinsic conditional autoregressive term.

**Table 3:** Negative binomial model Deviance Information Criterion (DIC) for each anthrome. Best models are shown in bold.

MODEL	DIC
CROPLAND	
**R + W + R:W + ICAR**	**128 354.10**
R + W + R:W	138 164.47
R + W + ICAR	128 374.22
R + W	138 166.24
RANGELAND	
R + W + R:W + ICAR	47 328.74
R + W + R:W	51 057.93
**R + W + ICAR**	**47 308.52**
R + W	51 035.93
SETTLED	
R + W + R:W + ICAR	29 799.93
R + W + R:W	32 597.90
R + W + ICAR	**29 784.36**
R + W	32 571.26
MIXED	
R + W + R:W + ICAR	52 781.57
R + W + R:W	58 049.01
**R + W + ICAR**	**52 780.20**
R + W	62 391.97
NATURAL	
R + W + R:W + ICAR	29 684.53
R + W + R:W	32 991.69
**R + W + ICAR**	**29 677.66**
R + W	32 981.13

To test the hypotheses that the weekly fire counts minima occur on Sunday (Friday) in Christian (Muslim) regions, we analyzed the pairwise contrasts Sunday *versus* every other week day in Christian regions, and Friday *versus* every other week day in Muslim regions. We also tested contrasts between fire counts on Sunday in Christian areas *versus* Friday in Muslim areas; on Sunday in Christian areas *versus* on Sunday in mixed religion areas; on Friday in Muslim areas *versus* on Friday in mixed areas (Table 4). The contrast analysis showed that, as hypothesized, there are significantly fewer fires on Sunday than on any other week day in Christian regions and on Friday than on any other week day in Muslim regions.

**Table 4:** Cropland anthrome estimated pairwise contrasts between Sunday (Friday) and every other week day in Christian (Muslim) regions, and between Sunday and Friday. The contrasts refer to the best cropland anthrome model shown in Table 3 (R + W + R:W + ICAR).

			95% credible interval			
Contrast	mean	s.d.	Lower	upper	p-value*	BH p-value**
Chr:Sun-Chr:Mon	-0.2113	0.0339	-0.2779	-0.1448	0.00000	0.00000
Chr:Sun-Chr:Tue	-0.2025	0.0338	-0.2689	-0.1361	0.00000	0.00000
Chr:Sun-Chr:Wed	-0.1204	0.0338	-0.1868	-0.0540	0.00036	0.00004
Chr:Sun-Chr:Thu	-0.1321	0.0339	-0.1986	-0.0657	0.00000	0.00000
Chr:Sun-Chr:Fri	-0.0996	0.0338	-0.1661	-0.0333	0.00319	0.00342
Chr:Sun-Chr:Sat	-0.1458	0.0339	-0.2123	-0.0794	0.00000	0.00000
Mus:Fri-Mus:Mon	-0.1807	0.0408	-0.2608	-0.1007	0.00000	0.00000
Mus:Fri-Mus:Tue	-0.2243	0.0408	-0.3044	-0.1444	0.00000	0.00000
Mus:Fri-Mus:Wed	-0.1985	0.0408	-0.2785	-0.1185	0.00000	0.00000
Mus:Fri-Mus:Thu	-0.2410	0.0408	-0.3211	-0.1610	0.00000	0.00000
Mus:Fri-Mus:Sat	-0.2104	0.0408	-0.2904	-0.1304	0.00000	0.00000
Mus:Fri-Mus:Sun	-0.1832	0.0408	-0.2632	-0.1032	0.00000	0.00000
Chr:Sun-Mus:Fri	0.5169	0.0906	0.3395	0.6951	0.00000	0.00000
Chr:Sun-Mix:Sun	0.6674	0.1374	0.4012	0.9403	0.00000	0.00000
Mus:Fri-Mix:Fri	0.0985	0.1718	-0.2362	0.4382	0.57856	0.57856

Chr: Christian; Mus: Muslim; Mon: Monday; Tue: Tuesday; Wed: Wednesday; Thu: Thursday; Fri: Friday; Sat: Saturday; Sun: Sunday.

* Probability that the posterior density function of the contrast is less than or equal to the posterior density function of the contrast at zero.

** Benjamini-Hochberg adjusted p-value.

## Discussion

Previous research based on remotely sensed data revealed different aspects of the anthropogenic nature of African vegetation burning. The very low interannual variability and strong annual periodicity of African fire activity, combined with the presence of a diurnal cycle were considered as evidence of markedly anthropogenic fire, caused by large-scale burning for land management purposes [34]. Anticipation of the fire season relatively to the dry season for periods of over one month throughout most of Africa was shown by [6], and interpreted as due to preventative break-up of fuel continuity at the landscape scale to avoid large, damaging fires, later in the season [7]. Statistical significance of the weekly cycle of fire activity in croplands, as opposed to the other anthromes, is understandable, considering that cropland fires typically are closely managed, small and low intensity. Less strictly controlled burning in natural areas and rangelands means that fires may be more intense [34], larger, and last longer than in agricultural areas [7,17]. Therefore, rangeland and forest fires observed by satellite on Friday (Sunday) in Muslim (Christian) regions are more likely to have been set in previous work days than the smaller, carefully controlled burns in fragmented agricultural landscapes, potentially reducing the difference between fire counts on holidays and week days. A study of preventative landscape burning in Mali [35] found that cropland fires were very well contained, with only a small number of farmers interviewed reporting that fires lit to prepare fields for agriculture had burned into the lands adjacent to their plots. Also in Mali, the occurrence of few, large fires in the northern pastoral areas of the Sahel, is opposed to a pattern of many small fires in agricultural south [7]. The same study refers smaller burns in croplands than in slash-and-burn farming in forest areas of Madagascar, while in southern Mozambique fires set to clear agricultural fields are smaller than those used to manage pastures [36].

Identification of a previously undetected weekly cycle in cropland burning further highlights the extent to which African fire is anthropogenic, and provides evidence that religion, although an essentially symbolic cultural system, has material impacts on the land surface that can be observed from space. Detailed geographical analysis of weekly cycles of fire activity will further elucidate the global geography and magnitude of anthropogenic vegetation burning, and may inform specification of the ignition component of fire modules incorporated in dynamic global vegetation models. Our detection of weekly cycles in vegetation burning contradicts the assumption of [14, 16] and lends support to the use of operational chemical weather forecasting models that rely on daily fire remote sensing data to estimate biomass burning emissions [37], which have important climatic effects [38,39] and impacts on public health [40]. Since biomass burning aerosols are strong absorbers of sunlight, they may also alter the thermodynamic structure of the atmosphere and contribute towards observed large scale weekly cycles in meteorological variables [41].
